# Bioactive Compounds and Biological Activities of *Jatropha curcas* L. Kernel Meal Extract

**DOI:** 10.3390/ijms12095955

**Published:** 2011-09-15

**Authors:** Ehsan Oskoueian, Norhani Abdullah, Syahida Ahmad, Wan Zuhainis Saad, Abdul Rahman Omar, Yin Wan Ho

**Affiliations:** 1 Department of Microbiology, Faculty of Biotechnology and Biomolecular Sciences, Universiti Putra Malaysia, 43400, Serdang, Selangor, Malaysia; E-Mails: ehs424@yahoo.com (E.O.); zuhainis@biotech.upm.edu.my (W.Z.S.); 2 Department of Biochemistry, Faculty of Biotechnology and Biomolecular Sciences, Universiti Putra Malaysia, 43400, Serdang, Selangor, Malaysia; E-Mail: syahida@biotech.upm.edu.my; 3 Institute of Tropical Agriculture, Universiti Putra Malaysia, 43400, Serdang, Selangor, Malaysia; 4 Institute of Bioscience, Universiti Putra Malaysia, 43400, Serdang, Selangor, Malaysia; E-Mails: aro@ibs.upm.edu.my (A.R.O.); ywho@ibs.upm.edu.my (Y.W.H.)

**Keywords:** *Jatropha curcas* L., bioactive compounds, antibacterial, antioxidant, cytotoxicity, kernel meal

## Abstract

Defatted *Jatropha curcas* L. (*J. curcas*) seed kernels contained a high percentage of crude protein (61.8%) and relatively little acid detergent fiber (4.8%) and neutral detergent fiber (9.7%). Spectrophotometric analysis of the methanolic extract showed the presence of phenolics, flavonoids and saponins with values of 3.9, 0.4 and 19.0 mg/g DM, respectively. High performance liquid chromatography (HPLC) analyses showed the presence of gallic acid and pyrogallol (phenolics), rutin and myricetin (flavonoids) and daidzein (isoflavonoid). The amount of phorbol esters in the methanolic extract estimated by HPLC was 3.0 *±* 0.1 mg/g DM. Other metabolites detected by GC-MS include: 2-(hydroxymethyl)-2 nitro-1,3-propanediol, β-sitosterol, 2-furancarboxaldehyde, 5-(hydroxymethy) and acetic acid in the methanolic extract; 2-furancarboxaldehyde, 5-(hydroxymethy), acetic acid and furfural (2-furancarboxaldehyde) in the hot water extract. Methanolic and hot water extracts of kernel meal showed antimicrobial activity against both Gram positive and Gram negative pathogenic bacteria (inhibition range: 0–1.63 cm) at the concentrations of 1 and 1.5 mg/disc. Methanolic extract exhibited antioxidant activities that are higher than hot water extract and comparable to β-carotene. The extracts tended to scavenge the free radicals in the reduction of ferric ion (Fe^3+^) to ferrous ion (Fe^2+^). Cytotoxicity assay results indicated the potential of methanolic extract as a source of anticancer therapeutic agents toward breast cancer cells.

## 1. Introduction

*Jatropha curcas* L. (*J. Curcas*) belongs to the *Euphorbiaceae* family and grows in tropical and sub-tropical regions like Central and South America, Africa, India and South East Asia [1]. It is a multipurpose plant with several industrial and medicinal applications. *Jatropha curcas* L. has been considered a potential source of seed oil for the production of biofuel. The plant ethnopharmacological applications are well known, but much of the information is empirical and lacking in scientific validation [2]. Terpenoid compounds are the major metabolites found in the Euphorbiaceae family. Among the terpenes, diterpenoids have dominated research in Jatropha species with respect to their novel chemical structures and medicinal values [3]. Recently, Oskoueian *et al.* [2] reported that extract of root and latex of *J. curcas* plant which contained phenolics, flavonoid and saponins showed notable antioxidant, anticancer and anti-inflammatory activities. These compounds have been reported to be involved in the biological activities of the plant [4].

Continuous efforts have been carried out to determine the presence of bioactive compounds in various plant materials, in particular, the agro-industrial by-products since they are renewable and abundantly available [4]. In the case of *J. curcas* kernel, the process of oil extraction produces a residue called kernel meal. This by-product has not been comprehensively studied, therefore limited information is available on the feasible applications of the meal as a potential source of bioactive compounds. Hence, the objective of the present study was to characterize the compounds present in the kernel meal and to investigate the biological activities of the meal extract. The information gathered would indicate the potential of the kernel meal as a source of bioactive compounds.

## 2. Results and Discussion

### 2.1. Chemical Composition

The chemical composition of kernel meal obtained after oil extraction was determined. As shown in Table 1, the meal is high in protein (61.8% w/w) but low in neutral detergent fiber (NDF) (9.7% w/w) and acid detergent fiber (ADF) (4.8% w/w). These results are comparable to those of *J. curcas* kernel meal from Mexico, India, Nicaragua and Cape Verde as reported by Makkar and Becker [1]. The phytochemical analyses of *J. curcas* kernel meal showed that total phenolics was 3.9 *±* 0.23 mg tannic acid equivalents/g DM and total flavonoid was 0.4 *±* 0.15 mg rutin equivalents/g DM (Table 1). Total phenolic content is comparable to the value of 3.6 mg tannic acid equivalents/g reported by Makkar and Becker [1], while total flavonoid content of kernel meal has not been reported so far.

Phenolic and flavonoid compounds occur ubiquitously in plants and a variety of biological activities have been attributed to them. These advantageous properties include antimicrobial, antiviral, anti-ulcerogenic, cytotoxic, anti-neoplastic, mutagenic, antioxidant, anti-hepatotoxic, anti-hypertensive, hypolipidemic, anti-platelet and anti-inflammatory activities. Many of these biological functions corresponded to their free radical scavenging and antioxidant activities [5].

Total saponin content was 19.0 *±* 0.48 mg diosgenin equivalents/g DM. This value was lower when compared to kernel meal of *J. curcas* from Mexico, Cape Verde and India with the values of 28.5, 26.0 and 27.3 mg diosgenin equivalents/g DM, respectively [6]. Saponins are widely distributed amongst plants and it is believed that saponins naturally act to protect the plant against pathogens. Their main biological activities include haemolytic, molluscicidal, anti-inflammatory, antimicrobial, anti-parasitic, cytotoxic and anti-tumor [7]. The presence of both polar (sugar) and nonpolar (steroid or triterpene) groups provide saponins with strong surface-active properties that are responsible for many of their biological activities such as antioxidant, antimicrobial, anti-inflammatory and cytotoxicity [8].

Phorbol esters are esters of tetra-cyclic diterpenes which are widely distributed in plant species of the families *Euporbiaceae* and *Thymelaceae* and biological activities such as anti-HIV, anti-malaria and antimicrobial have been reported by Goel *et al.* [9]. The phorbol esters content of kernel meal was 3.0 *±* 0.16 mg phorbol-12-myristate 13-acetate (PMA) equivalents/g DM. Phorbol esters are known to mimic the action of diacylglycerol to activate the protein kinase C, which regulates different signal transduction pathways and other cellular metabolic activities including proliferation, malignant transformation, differentiation and cell death [9]. The multiplicity of effects of phorbol esters on biological systems is associated with the presence of phorbol esters receptors [10] and the types of phorbol esters. Fujii *et al.* [10] and Park *et al.* [11] reported the induction of apoptosis using phorbol esters in prostate cancer cells and gastric cancer cells.

### 2.2. Phenolics and Flavonoids Analyses by HPLC

**Figure 1:**
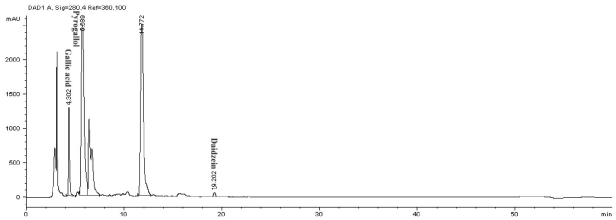
HPLC chromatogram of phenolic and isoflavonoid compounds from methanolic extract of *J. curcas* kernel meal detected at 280 nm.

**Figure 2:**
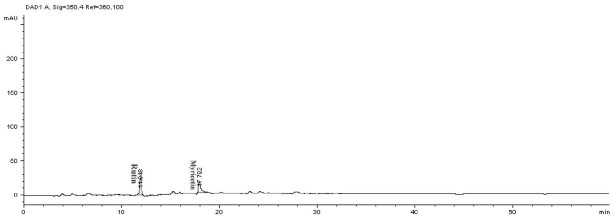
HPLC chromatogram of flavonoid compounds from methanolic extract of *J. curcas* kernel meal detected at 350 nm.

In this study, high performance liquid chromatography (HPLC) was used to determine the phenolic and flavonoid compounds in *J. curcas* kernel meal extracts. The analyses showed the presence of gallic acid and pyrogallol as phenolics (Figure 1); rutin and myricetin as flavonoids (Figure 2) and daidzein as isoflavonoid compound (Figure 1) with the concentrations shown in Table 2. Compounds that have been reported from *J. curcas* leaves including apigenin and its glycosides, vitexin and isovitexin, stigmasterol, β-sitosterol and gallic acid [12] while the root and stem contained gallic acid, ellagic acid, quercetin, coumaric acid, benzoic acid and salicylic acid [13].

### 2.3. Metabolite Composition Analysis by GC-MS

The GC-MS analysis is a rapid and simple method to determine the compounds present in the plant materials. Table 3 shows the major organic compounds detected by GC-MS in the methanolic and hot water extracts. More than half of the organic compounds in the methanolic extract consists of 2-(hydroxymethyl)-2 nitro-1,3-propanediol, β-sitosterol, 2-furancarboxaldehyde, 5-(hydroxymethy) and acetic acid. In the hot water extract, the most common compounds present are 2-furancarboxaldehyde, 5-(hydroxymethy), acetic acid and furfural (2-furancarboxaldehyde) adding up to about 60% of the total extracted materials. The 2-(Hydroxymethyl)-2 nitro-1,3-propanediol has been reported as microbicidal and it is used as a bacteriostat in disinfectants [14]. β-sitosterol, a plant sterol, shows antimicrobial [15], anti-inflammatory and cytotoxic activities [16,17]. The compound 2-furancarboxaldehyde, 5-(hydroxymethy) also shows antibacterial and antifungal activity applicable in pharmaceutical, cosmetics and pesticides [18]. Furfural is used as a flavor in foods, and in other products, such as cosmetics, fragrance, pesticide, herbicide, fungicide, insecticide and germicide [19]. According to Ryssel *et al.* [20], acetic acid also showed excellent bactericidal effects toward *Proteus vulgaris*, *Pseudomonas aeruginosa* and *Acinetobacter baumannii*. Therefore, the GC-MS results showed the presence of compounds with known activities indicating the *J. curcas* kernel meal as a potential source of functional metabolites.

### 2.4. Antibacterial Activity

Antibacterial activity of methanolic and hot water extracts of *J. curcas* kernel meal were tested against four Gram negative and four Gram positive pathogens at two concentrations (1.0 and 1.5 mg/disc). The results shown in Table 4 indicate significant difference (*P* < 0.05) in inhibitory activity of methanolic and hot water extracts. The diameter of inhibition zones of methanolic extract ranged from 0.35 to 1.25 cm while for hot water extract ranged from 0.00 to 0.75 cm at 1.0 mg/disc. Antimicrobial activity of the methanolic extract appeared to be more effective than water extract, since methanol could extract a wide variety of active components compared to hot water. The size of inhibition zones increased with 1.5 mg/disc. At this concentration, the antibacterial effect of methanolic extract was similar for both Gram negative and Gram positive bacteria, except for *P. aeruginosa*, which showed moderate zone of inhibition. This Gram negative bacteria seemed to be less sensitive to other plant extracts [21].

*Escherichia coli* seemed to be the most susceptible bacterial species to methanolic and hot water extracts at 1.0 and 1.5 mg/disc extract concentration. The antibacterial activities of both kanamycin and streptomycin were as expected and some of the clearing zones of pathogens were comparable to that of antibiotics, particularly when 1.5 mg/disc extracts were tested.

Various reports have shown the antimicrobial activity of extracts from different parts of *J. curcas* plant. However, the efficacy of extracts to inhibit microbial growth seemed to vary according to extraction procedures and assay methods. Igbinosa *et al.* [22] have reported the *in vitro* antimicrobial activity of crude methanolic and water extracts of the *J. curcas* stem bark against a wide spectrum of microorganisms with zones of inhibition ranging from 0.8 to 2 and 0 to 0.8 cm for methanol and water extracts at the concentration of 1.0 mg per well (0.6 mm) respectively. In another study, Atindehou *et al.* [23] reported the antimicrobial activity of the leaves and roots of *J. curcas* against *E. coli*, *P. aeruginosa*, *S. aureus* and *E. faecalis*.

Total phenolics generally possess antimicrobial activities which provide chemical barriers for invading microorganisms. On the other hand, flavonoids inhibit bacterial growth by inhibition of DNA gyrase, cytoplasmic membrane function and energy metabolism [24]. Gallic acid and pyrogallol as phenolic compounds, daidzein as isoflavonoid and rutin and myricetin as flavonoids (Table 2) are substantially effective as antibacterial agent [24]. Additionally, the saponins present in *J. curcas* kernel meal could also contributed to the antimicrobial activity as saponins have been shown to possess antibacterial activity against several Gram positive and Gram negative bacteria [7]. Furthermore, phorbol esters (Table 1) could also act as antimicrobial agent as this property has been demonstrated by Chumkaew *et al.* [25].

### 2.5. Antioxidant Activity

**Figure 3:**
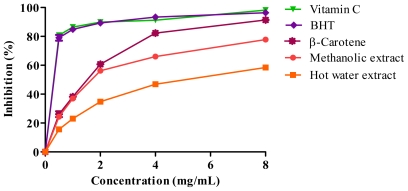
The free radical scavenging activity of *J. curcas* kernel meal extracts and reference antioxidants.

The results shown in Figure 3 indicate the 2,2-diphenyl-1-picrylhydrazyl (DPPH) radical scavenging activity of *J. curcas* kernel meal extracts at different concentrations. The inhibition of DPPH radical scavenging activity increased in a dose-dependent manner. Table 5 shows the IC_50_ (required concentration to scavenge 50% of DPPH radicals) concentration of extracts. Free radical scavenging activity of methanolic extract showed a value of 1.6 mg/mL but hot water extract did not show an IC_50_ value, even at the highest concentration used in this study. Methanolic extract of *J. curcas* kernel meal appeared to be more active as compared to the hot water extract in scavenging the free radicals. Reference antioxidants vitamin C, BHT and β-carotene showed IC_50_ values of 0.3, 0.3 and 1.5 mg/mL respectively. Both extracts showed lower activities than vitamin C and BHT due to the significantly (*p* < 0.05) higher IC_50_ values required to inhibit the free radicals (Table 5). The methanolic extract activity was similar to β-carotene as no significant difference was observed between their IC_50_ values (Table 5). It has been reported that the strong free radical scavenging activities in latex, root and stem of *J. curcas* was attributed to the presence of phenolic compounds [2,13].

**Figure 4:**
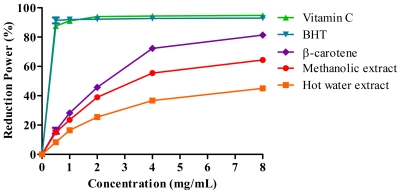
Ferric reducing activity of *J. curcas* kernel meal extracts and reference antioxidants.

Ferric reducing antioxidant power (FRAP) of extracts and reference antioxidants (vitamin C, BHT and β-carotene) is presented in Figure 4. Reference antioxidants showed higher reducing power followed by methanolic and hot water extract. The IC_50_ value (the concentration to reduce 50% of Fe^3+^ to Fe^2+^ as an index for antioxidant potential) in FRAP for vitamin C, BHT and β-carotene were 0.3, 0.3 and 2.6 mg/mL, respectively (Table 5), while for the methanolic and hot water extracts were 3.0 and 8.0 mg/mL, respectively. As observed in the DPPH assay, FRAP assay also showed similar IC_50_ values between methanolic extract and β-carotene (Table 5).

The results indicate that the extracts were more active in scavenging free radicals compared to ferric reducing power activity. In FRAP assay, the general ability of the extracts to donate electrons is tested whereas, in the DPPH assay, reduction by hydrogen atoms are also involved. Hence, the free radical scavenging activity assay was a better indicator of antioxidant activity of kernel meal than FRAP based on the reaction mechanisms proposed. In complex systems, such as food, various different mechanisms may contribute to oxidative processes. Therefore, it is important to characterize the extracts by a variety of antioxidant assays [26].

The presence of phenolics (gallic acid, pyrogallol), flavonoids (rutin, myricetin) and isoflavonoid (daidzein) in the kernel meal (Table 2) may contribute to the antioxidant activity of extracts. These compounds have been identified as some of the major compounds involved in antioxidant activity [27]. However, saponins in the kernel meal (Table 1) might be also involved, because Sparg *et al.* [7] reported the antioxidant activity of these compounds.

### 2.6. Cytotoxicity Activity

**Figure 5:**
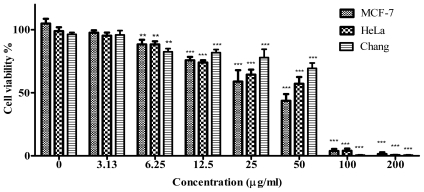
Effect of methanolic extract of *J. curcas* kernel meal on MCF-7, HeLa and Chang liver cell viabilities. All bars represent the mean *±* standard error of three independent experiments. *** *P* < 0.0001 and ** *P* < 0.001 indicates significant difference compared to the untreated control group.

Phytochemical analyses, antimicrobial and antioxidant activities showed that the methanolic extract was more active compared to hot water extract. Therefore, cytotoxicity assay was performed using the methanolic extract. It is known that different cell lines might exhibit different sensitivities while treated with the same plant extract, therefore screening plant crude extract against various cell lines is a first step in determining the most susceptible cancer cells. Apart from that, breast and cervical cancers are very common manifestations, hence the MCF-7 and HeLa cell lines were used to investigate the effects of the plant extract. Chang liver cells (human hepatocytes) were also used to determine the hepatoxicity of extract used in this study. Cytotoxicity assay results demonstrated that the methanolic extract of *J. curcas* kernel meal inhibited the growth and proliferation of MCF-7, HeLa and Chang liver cells in a dose-dependent manner (Figure 5). Methanolic extract at the concentration of 6.25 μg/mL and above decreased the cell viabilities significantly (*P* < 0.0001) in all cell lines used in this study as compared to the untreated control.

**Figure 6:**
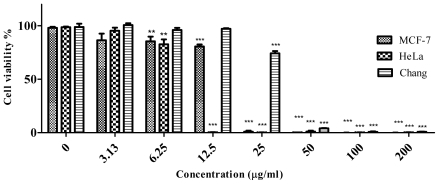
Effect of Tamoxifen as a positive control on MCF-7, HeLa and Chang liver cell viabilities. All bars represent the mean *±* standard error of three independent experiments. *** *P* < 0.0001 and ** *P* < 0.001 indicates significant difference compared to the untreated control group.

In this cytotoxicity activity study, Tamoxifen was used as a positive control. As shown in Figure 6, Tamoxifen also inhibited the growth and proliferation of MCF-7, HeLa and Chang liver cells in a dose dependent manner. At 6.25 μg/mL and above, cell viabilities decreased significantly (*P* < 0.0001) in all cell lines when compared to the untreated control.

The CC_50_ values of methanolic extract and Tamoxifen are presented in Table 6. The MCF-7 cells were found to be more susceptible to the methanolic extract as compared to the HeLa and Chang cells. Most anticancer drugs have been discovered through random screening of plant materials. The National Cancer Institute requires a CC_50_ value of less than 30 μg/mL for the active crude extracts against cancer cells [28]. Since the methanolic extract showed CC_50_ values less than National Cancer Institute requirement toward MCF-7 cells, methanolic extract could be a promising source of bioactive compounds that could be purified for the development of anticancer drugs in order to treat breast cancer.

The CC_50_ concentrations of Tamoxifen (Table 6) for MCF-7, HeLa and Chang liver cell lines were 17.3, 8.89, 33.6 μg/mL, respectively. The activity of Tamoxifen toward MCF-7, HeLa and Chang were significantly (*p* < 0.0001) higher than methanolic extract. However, normal Chang liver cells were also susceptible to both methanolic extract and Tamoxifen. It is interesting to note that the CC_50_ concentration of Tamoxifen was about half of that methanolic extract. At present it is not known which particular compound(s) present in the kernel meal methanolic extract exert the cytotoxicity effect in both cancer and, to some extent, Chang liver cells.

The presence of bioactive compounds in other *J. curcas* plant parts has been reported recently. Oskoueian *et al.* [2] demonstrated the cytotoxic effect of *J. curcas* root and latex methanolic extract with CC_50_ values of 18.3 *±* 0.98 and 70.1 *±* 2.06 μg/mL against HT-29 cloned cancer cells, respectively; and 33.3 *±* 0.75 and 60.1 *±* 0.89 μg/mL toward Chang liver cells, respectively. Besides root and latex, leaves methanolic extract was also observed to be effective against B16F10 melanoma cells with CC_50_ value of 24.8 μg/mL [29]. Moreover, Fujii *et al.* [10] reported that PMA induced apoptosis in prostate cancer cells through overexpression of PKCδ. Park *et al.* [11] also concluded that the PMA induce apoptosis in gastric cancer cells through activation of PKC and the activation of serine protease(s) and caspase-3/CPP32. All these results indicate the potential of *J. curcas* plant as a source of bioactive compounds, but, the exact nature of the metabolite involved has to be elucidated, although it has been reported that phenolics, flavonoids, saponins and phorbol esters have been shown to be cytotoxic on different cell lines [9].

## 3. Experimental Section

### 3.1. Sample and Extract Preparation

The *Jatropha curcas* L. plant was collected from the farm of Faculty of Agriculture, Universiti Putra Malaysia with the GPS location of 3°0′26.91″N latitude and 101°42′13.24″E longitude for identification by Mr. Shamsul Khamis. A voucher specimen (SK1764/2010) was deposited in the Phytomedicinal Herbarium, Institute of Bioscience, Universiti Putra Malaysia, Serdang, Selangor, Malaysia. Upon confirmation of the plant, the ripe *J. curcas* seeds were collected from the farm, air dried, dehulled and kept at 4 °C for further analysis. The kernels isolated were ground, using a mechanical grinder and oil was extracted by Soxhlet apparatus, using petroleum ether (boiling point of 40–60 °C) for 16 h [30]. Methanolic extract of *J. curcas* kernel meal was prepared according to Crozier *et al.* [27]. Hot water extract was prepared as described by Gulcin *et al.* [31]. The extracts were filtered through Whatman No.1 paper and evaporated by using vacuum rotary evaporator (Buchii, Switzerland). The crude extracts were re-dissolved in methanol for antioxidant, antimicrobial and HPLC analyses, or in dimethyl sulfoxide for cytotoxicity assay. The extractions were repeated three times.

### 3.2. Chemical Constituents

Chemical constituent analyses were conducted according to AOAC [32]. The fiber fractions which consisted of neutral detergent fiber (NDF) and acid detergent fiber (ADF) were determined according to Van Soest *et al.* [33]. The analyses were repeated three times.

### 3.3. Phytochemical Analyses

#### 3.3.1. Total Phenolics

Total phenolics was determined by using Folin–Ciocalteu reagent according to Ismail *et al.* [26] and total phenolic results were expressed as mg tannic acid equivalentss/g DM of the kernel meal. The analysis was conducted in triplicate.

#### 3.3.2. Total Flavonoids

Total flavonoids was determined based on aluminium chloride colorimetric assay described by Ismail *et al.* [26]. Total flavonoid compound of extracts was expressed as mg rutin equivalents/g DM of the kernel meal. The analysis was conducted in triplicate.

#### 3.3.3. Total Saponins

Total saponin was determined according to Makkar *et al.* [8] based on vanillin-sulfuric acid colorimetric reaction and results expressed as mg diosgenin equivalents/g DM of the kernel meal. The analysis was conducted in triplicate.

### 3.4. Analyses of Phenolic and Flavonoid Compounds by HPLC

The phenolic and flavonoid compounds of *J. curcas* kernel meal were quantitatively measured by HPLC based on the method described by Crozier *et al.* [27] with some modification. Phenolic standards were gallic acid, syringic acid, vanillic acid, salicylic acid, caffeic acid and pyrogallol. Flavonoid standards used in this research were quercetin, rutin, myricetin, kaempferol, naringin and apigenin and isoflavonoid standards were genistein and daidzein. An aliquot of sample extracts was loaded on the HPLC equipped with an analytical column Intersil ODS-3 (5 μm 4.6 × 150 mm, Gl Science Inc.). Solvents comprising deionized water (solvent A) and acetonitrile (solvent B) were used. The pH of water was adjusted to 2.5 with trifluoroacetic acid. The phenolic and isoflavonoid compounds were detected at 280 nm while flavonoid compounds at 350 nm. The column was equilibrated by 85% solvent A and 15% solvent B. Then the ratio of solvent B was increased to 85% in 50 min followed by reducing solvent B to 15% in 55 min. This ratio was maintained for 60 min for each sample analysis with flow rate at 0.6 mL/min. The analysis was repeated three times.

### 3.5. Analysis of Phorbol Esters

Phorbol esters were determined based on Makkar *et al.* [8]. An aliquot of sample extracts was loaded on a high-performance liquid chromatography (HPLC Agilent-1200) series instrument equipped with a UV-Vis photodiode array (DAD) detector, binary pump, vacuum degasser, auto sampler and analytical column Agilent Reverse-phase C_18_ LiChrospher 100 (250 × 4 mm I.D and 5 μm pore size). The solvents were deionized water (solvent A) and acetonitrile (solvent B). The absorbance was read at 280 nm and results were expressed as equivalents to phorbol-12-myristate 13-acetate standard. The analysis was done in triplicate.

### 3.6. Analysis of Metabolites by Gas Chromatography-Mass Spectrometry (GC-MS)

The chemical composition of the extracts was characterized by GC-MS based on the method described by Hossain and Rahman [34]. The GC-MS used was a Schimadzu QP2010PLUS system. Six μL were analysed on a BPX-5 SGE ultra-low-bleed 5% phenyl polydimethylsiloxane capillary column (30 m × 0.25 mm i.d. × 0.25 μm film thickness). Splitless injection was performed with a purge time of 1.0 min. The carrier gas was helium at a flow rate of 1 mL min^−1^. The column temperature was maintained at 50 °C for 3 min, then programmed at 5°C min^−1^ to 80 °C and then at 10 °C min^−1^ to 340 °C. The inlet and detector temperature was 250 °C and 340 °C, respectively, and the solvent delay was 4 min. The identification of the peaks was based on computer matching of the mass spectra with the National Institute of Standards and Technology (NIST 08 and NIST 08s) library and by direct comparison with published data. The analyses were carried out in triplicate.

### 3.7. Antibacterial Activity

The antibacterial assay of *J. curcas* kernel meal extracts was carried out by the disc diffusion method as described by Boussaada *et al.* [21] against Gram positive (*Micrococcus luteus*, *Bacillus subtilis* B145, *Bacillus cereus* B43 and *Staphylococcus aureus* S1431) and Gram negative (*Enterobacter aerogenes*, *Klebsiella pneumonia* K36, *Escherichia coli* E256 and *Pseudomonas aeruginosa* PI96) pathogens. All the bacteria were purchased from the Institute of Malaysian Research (IMR) and maintained in the Department of Microbiology, Faculty of Biotechnology and Biomolecular Sciences, Universiti Putra Malaysia. The positive control without extracts (solvent) and reference controls using two standard antibiotics (kanamycin and streptomycin) were included in the assay. The extracts inhibitions were corrected based on positive control and compared with those of reference control. The experiments were conducted in triplicate.

### 3.8. Antioxidant Activity

#### 3.8.1. DPPH Radical-Scavenging Activity

Free radical scavenging activity of extracts was determined with 1,1-diphenyl-2-picryl-hydrazil (DPPH) as free radicals according to Ismail *et al.* [26]. The absorbance was measured at 515 nm by using a spectrophotometer. Vitamin C, butylated hydroxytoluene (BHT) and β-carotene were used as standard antioxidants. The assay was performed in triplicate.

#### 3.8.2. Ferric-Reducing Antioxidant Power (FRAP) Assay

The ferric reducing antioxidant power (FRAP) of the extracts was determined as described by Yen and Chen [35]. Vitamin C, butylated hydroxytoluene (BHT) and β-carotene were used as standard antioxidants. The assay was performed in triplicate.

### 3.9. Cytotoxicity Assay

Two cancer cell line, including human breast cancer cell line (MCF-7 ATCC: HTB-22) and human cervical adenocarcinoma (HeLa ATCC: CCL-2) and one normal cell line, human hepatocytes (Chang ATCC: CCL-13) were purchased from the American Type Culture Collection (ATCC). Cell lines were grown as monolayers in a T-75 cm^2^ culture flask. The Dulbecco’s Modified Eagle Medium (DMEM) was supplemented with 2.0 g/L sodium bicarbonate and 10% fetal bovine serum. The cultured cell lines were maintained in a humidified atmosphere of 5% CO_2_ at 37 °C and were harvested when they reached 80% confluency for the cytotoxic test. Cytotoxicity of *J. curcas* kernel meal extract was measured by MTT cell proliferation assay [36]. Cytotoxic concentration was expressed as CC_50_ (concentration of the sample that decreased the number of viable cells to 50% of the control cell) which was calculated by Graph Pad Prism 5 software. All determinations were performed in triplicate.

### 3.10. Statistical Analyses

Inhibition zones of antibacterial assay subjected to the general linear models procedure of SAS [37] in a completely randomized design and the means were compared with Duncan’s Multiple Range test. The difference was considered significant when the *P* value was <0.05. GraphPad Prism 5 software (GraphPad Software Inc., San Diego, CA) was used for all the statistical analyses in cytotoxic assay.

## 4. Conclusions

The kernel meal obtained from *J. curcas* seeds grown in Malaysia contains high percentage of crude protein and relatively low fiber. Analysis by HPLC showed the presence of various phenolics and flavonoids, together with saponins and phorbol esters, while GC-MS analyses indicated the presence of other metabolites that are probably involved in the antibacterial, antioxidant and cytotoxicity activities observed in this study. The ability of methanolic extract to inhibit breast cancer cell growth indicates the potential value of *J. curcas* kernel meal as an alternative source of therapeutic agents which requires further investigation.

## Figures and Tables

**Table 1:** Chemical and phytochemical analyses of *J. curcas* kernel meal (on dry matter basis) *.

Chemical Analyses (%)		Phytochemical Analyses (mg/g DM)	
Dry Matter (DM)	94.2 ± 0.53	Total phenolic c	3.9 ± 0.23
Crude Protein	61.8 ± 0.11	Total flavonoid d	0.4 ± 0.15
Organic Matter	91.6 ± 0.04	Total saponin e	19.0 ± 0.48
Ash	8.3 ± 0.07	Total phorbol esters f	3.0 ± 0.16
NDF a	9.7 ± 0.52		
ADF b	4.8 ± 0.21		
Gross Energy (MJ/kg)	17.7 ± 0.19		

a Neutral detergent fiber;

b Acid detergent fiber;

c Tannic acid equivalents;

d Rutin equivalents;

e Diosgenin equivalents;

f Equivalent to phorbol 12-myristate,13 acetate;

* Each value represents the mean ± S.E.M of three replicates.

**Table 2:** Concentrations of phenolic, flavonoid and isoflavonoid compounds in *J. curcas* kernel meal methanolic extract (μg/g) *.

Sample	Phenolic (μg/g)		Flavonoid (μg/g)		Isoflavonoid (μg/g)
	Gallic Acid	Pyrogallol	Rutin	Myricetin	Daidzein
*J. curcas* kernel meal	581 ± 0.4	631 ± 0.5	48 ± 0.5	199 ± 0.3	298 ± 0.3

All values are on DM basis;

* Each value represents the mean ± S.E.M of three replicates.

**Table 3:** Major organic compounds detected in the methanolic and hot water extracts of *J. Curcas* kernel meal.

Methanolic Extract		Hot Water Extract	
Compounds	Content (% w/w)	Compounds	Content (% w/w)
2-(Hydroxymethyl)-2 nitro-1,3-propanediol	23.1	2-Furancarboxaldehyde, 5-(hydroxymethy)	29.7
β-sitosterol	13.8	Acetic acid	16.2
2-Furancarboxaldehyde, 5-(hydroxymethy)	8.9	Furfural (2-furancarboxaldehyde)	14.6
Acetic acid	10.7		

**Table 4:** Inhibition zones of *J. curcas* kernel meal extracts against pathogenic bacteria at the concentration of 1.0 and 1.5 mg/disc.

Microorganism	Zone of Inhibition (cm)						
	Methanol		Hot Water	Kanamycin		Streptomycin	
	
	1.0 mg/disc	1.5 mg/disc	1.0 mg/disc	1.5 mg/disc	1.0 μg/disc	1.0 μg/disc	S.E.M *
**Gram negative**							
*Enterobacter aerogenes*	1.11 ^b^	1.21 ^b^	0.21 ^e^	0.60 ^d^	1.54 ^a^	0.96 ^c^	0.04
*Klebsiella pneumoniae*	1.05 ^c^	1.20 ^b^	0.40 ^e^	0.62 ^d^	1.44 ^a^	1.04 ^c^	0.02
*Escherichia coli*	1.25 ^c^	1.63 ^b^	0.75 ^e^	1.00 ^d^	1.70 ^a^	1.03 ^d^	0.02
*Pseudomonas aeruginosa*	0.35 ^d^	0.58 ^b^	0.45 ^c^	0.37 ^c,d^	1.01 ^a^	0.41 ^c,d^	0.02
**Gram positive**							
*Micrococcus luteus*	0.5 ^c^	1.20 ^a^	0.22 ^e^	0.30 ^d^	1.35 ^b^	1.10 ^b^	0.02
*Bacillus subtilis*	1.10 ^b^	1.40 ^a^	0.65 ^d^	0.85 ^c^	1.23 ^b^	0.86 ^c^	0.04
*Bacillus cereus*	1.00 ^d^	1.25 ^b^	0.00 ^f^	0.77 ^e^	1.49 ^a^	1.07 ^c^	0.02
*Staphylococcus aureus*	1.15 ^b^	1.37 ^a^	0.30 ^e^	0.75 ^d^	1.33 ^a^	0.95 ^c^	0.04

Means with different superscripts within rows are significantly different (*P* < 0.05); Analyses were done in triplicate;

* S.E.M: Standard error of mean.

**Table 5:** The IC_50_ values of *J. curcas* kernel meal extracts and reference antioxidants.

Items	IC_50_ (mg/mL)	
	DPPH	FRAP
Methanolic extract	1.6 ^b^	3.0 ^b^
Hot water extract	5.0 ^a^	>8 ^a^
Vitamin C	0.3 ^c^	0.3 ^c^
BHT	0.3 ^c^	0.3 ^c^
β-carotene	1.5 ^b^	2.6 ^b^

* S.E.M	0.09	0.07

*P* value	0.05	0.05

Values with different subscripts in each column are significantly different (*p* < 0.05); Analyses were done in triplicate;

* S.E.M: Standard error of mean.

**Table 6:** The CC_50_ values of extracts and positive control (Tamoxifen) against MCF-7, HeLa and Chang liver cell lines.

Sample	CC_50_ value (μg/mL)		
	MCF-7	HeLa	Chang liver cell
Methanolic extract	27.5 ^a^	56.4 ^a^	63.9 ^a^
Tamoxifen	17.3 ^b^	8.89 ^b^	33.6 ^b^

* S.E.M	0.35	0.42	0.64

*P* value	0.0001	0.0001	0.0001

Analyses were done in triplicate;

* S.E.M: Standard error of mean.

## References

[1] Makkar H, Becker K (2009). *Jatropha curcas*, a promising crop for the generation of biodiesel and value-added coproducts. Eur J Lipid Sci Technol.

[2] Oskoueian E, Abdullah N, Saad WZ, Omar AR, Ahmad S, Kuan WB, Zolkifli NA, Hendra R, Ho YW (2011). Antioxidant, anti-inflammatory and anticancer activities of methanolic extracts from *Jatropha curcas* Linn. J Med Plants Res.

[3] Devappa RK, Makkar HPS, Becker K (2011). Jatropha Diterpenes: a Review. J Am Oil Chem Soc.

[4] Balasundram N, Sundram K, Samman S (2006). Phenolic compounds in plants and agri-industrial by-products: Antioxidant activity, occurrence, and potential uses. Food Chem.

[5] Muraoka S, Miura T (2004). Inhibition of xanthine oxidase by phytic acid and its antioxidative action. Life Sci.

[6] Devappa RK, Swamylingappa B (2008). Biochemical and nutritional evaluation of *Jatropha* protein isolate prepared by steam injection heating for reduction of toxic and anti nutritional factors. J Sci Food Agric.

[7] Sparg SG, Light ME, Van Staden J (2004). Biological activities and distribution of plant saponins. J Ethnopharmacol.

[8] Makkar HPS, Siddhuraju P, Becker K (2007). Methods in Molecular Biology: Plant Secondary Metabolites.

[9] Goel G, Makkar HPS, Francis G, Becker K (2007). Phorbol esters: structure, biological activity, and toxicity in animals. Int J Toxicol.

[10] Fujii T, Garcia-Bermejo ML, Bernabo JL, Caamano J, Ohba M, Kuroki T, Li L, Yuspa SH, Kazanietz MG (2000). Involvement of protein kinase C (PKC) in phorbol ester-induced apoptosis in LNCaP prostate cancer cells. J Biol Chem.

[11] Park IC, Park MJ, Rhee CH, Lee JI, Choe TB, Jang JJ, Lee SH, Hong SI (2001). Protein kinase C activation by PMA rapidly induces apoptosis through caspase-3/CPP32 and serine protease(s) in a gastric cancer cell line. Int J Oncol.

[12] Chhabra SC, Mahunnah RLA, Mshiu EN (1990). Plants used in traditional medicine in Eastern Tanzania. III. Angiosperms (Euphorbiaceae to Menispermaceae). J Ethnopharmacol.

[13] El Diwani G, El Rafie S, Hawash S (2009). Antioxidant activity of extracts obtained from residues of nodes leaves stem and root of Egyptian *Jatropha curcas*. Afr J Pharm Pharacol.

[14] Popendorf W, Selim M, Lewis MQ (1995). Exposure while applying industrial antimicrobial pesticides. Am Ind Hyg Assoc J.

[15] Ajaiyeoba EO, Onocha PA, Nwozo SO, Sama W (2003). Antimicrobial and cytotoxicity evaluation of *Buchholzia coriacea* stem bark. Fitoterapia.

[16] Gupta MB, Nath R, Srivastava N, Shanker K, Kishor K, Bhargava KP (1980). Anti-inflammatory and antipyretic activities of β-sitosterol. Planta Med.

[17] Awad A, Chen YC, Fink C, Hennessey T (1996). Sitosterol inhibits HT-29 human colon cancer cell growth and alters membrane lipids. Anticancer Res.

[18] Rigal L, Gaset A (1983). Direct preparation of 5-hydroxymethyl-2-furancarboxaldehyde from polyholosides: a chemical valorisation of the Jerusalem artichoke (*Helianthus tuberosus* L.). Biomass.

[19] Morales FJ, Stadler RH, Lineback DR (2008). Hydroxymethylfurfural (HMF) and Related Compounds. Process-Induced Food Toxicants.

[20] Ryssel H, Kloeters O, Germann G, Schafer T, Wiedemann G, Oehlbauer M (2009). The antimicrobial effect of acetic acid-An alternative to common local antiseptics?. Burns.

[21] Boussaada O, Chriaa J, Nabli R, Ammar S, Saidana D, Mahjoub M, Chraeif I, Helal A, Mighri Z (2008). Antimicrobial and antioxidant activities of methanol extracts of *Evax pygmaea* (Asteraceae) growing wild in Tunisia. World J Microb Biot.

[22] Igbinosa O, Igbinosa E, Aiyegoro O (2009). Antimicrobial activity and phytochemical screening of stem bark extracts from *Jatropha curcas* (Linn). Afr J Pharm Pharacol.

[23] Atindehou K, Kone M, Terreaux C, Traore D, Hostettmann K, Dosso M (2002). Evaluation of the antimicrobial potential of medicinal plants from the Ivory Coast. Phytother Res.

[24] Cushnie T, Lamb A (2005). Antimicrobial activity of flavonoids. Int J Antimicro Ag.

[25] Chumkaew P, Karalai C, Ponglimanont C, Chantrapromma K (2003). Antimycobacterial activity of phorbol esters from the fruits of Sapium indicum. J Nat Prod.

[26] Ismail HI, Chan KW, Mariod AA, Ismail M (2010). Phenolic content and antioxidant activity of cantaloupe (*cucumis melo*) methanolic extracts. Food Chem.

[27] Crozier A, Lean M, McDonald M, Black C (1997). Quantitative analysis of the flavonoid content of commercial tomatoes, onions, lettuce, and celery. J Agric Food Chem.

[28] Boik JC (2001). Natural Compounds in Cancer Therapy.

[29] Balaji R, Rekha N, Deecaraman M, Manikandan L (2009). Antimetastatic and antiproliferative activity of methanolic fraction of *Jatropha curcas* against B16F10 melanoma induced lung metastasis in C57BL/6 mice. Afr J Pharm Pharacol.

[30] Martinez-Herrera J, Siddhuraju P, Francis G, Davila-Ortiz G, Becker K (2006). Chemical composition, toxic/antimetabolic constituents, and effects of different treatments on their levels, in four provenances of *Jatropha curcas* L. from Mexico. J Food Chem.

[31] Gulcin I, Gungor Sat I, Beydemir S, Elmastas M, Irfan Kufrevioglu O (2004). Comparison of antioxidant activity of clove (*Eugenia caryophylata* Thunb) buds and lavender (*Lavandula stoechas* L.). Food Chem.

[32] AOAC (1990). Official Methods of Analysis.

[33] Van Soest PJ, Robertson JB, Lewis BA (1991). Methods for dietary fiber, neutral detergent fiber, and nonstarch polysaccharides in relation to animal nutrition. J Dairy Sci.

[34] Hossain M, Rahman A (2011). Chemical composition of bioactive compounds by GC-MS screening and anti-fungal properties of the crude extracts of cabbage samples. Asian J Biotechnol.

[35] Yen G, Chen H (1995). Antioxidant activity of various tea extracts in relation to their antimutagenicity. J Agric Food Chem.

[36] Sharif R, Ghazali A, Rajab N, Haron H, Osman F (2008). Toxicological evaluation of some Malaysian locally processed raw food products. Food Chem Toxicol.

[37] SAS (2003). SAS, Statistical Analysis Institute (Version 9.1.3).

